# Whole-Milk Dairy Foods: Biological Mechanisms Underlying Beneficial Effects on Risk Markers for Cardiometabolic Health

**DOI:** 10.1016/j.advnut.2023.09.001

**Published:** 2023-09-07

**Authors:** Moises Torres-Gonzalez, Beth H. Rice Bradley

**Affiliations:** 1 Nutrition Research, National Dairy Council, Rosemont, IL, United States; 2 Department of Nutrition and Food Sciences, University of Vermont, Burlington, VT, United States

**Keywords:** dairy, milk, cheese, yogurt, food matrix, cardiovascular disease, metabolic health, milk fat globular membrane, milk polar lipids, fermented dairy

## Abstract

Lifestyle modifications that include adherence to healthy dietary patterns that are low in saturated fat have been associated with reduced risk for cardiovascular disease, the leading cause of death globally. Whole-milk dairy foods, including milk, cheese, and yogurt, are leading sources of saturated fat in the diet. Dietary guidelines around the world recommend the consumption of low-fat and fat-free dairy foods to obtain overall healthy dietary patterns that help meet nutrient recommendations while keeping within recommended calorie and saturated fat limitations. A body of observational and clinical evidence indicates, however, that whole-milk dairy food consumption, despite saturated fat content, does not increase the risk for cardiovascular disease. This review describes the proposed biological mechanisms underlying inverse associations between whole-milk dairy food consumption and risk markers for cardiometabolic health, such as altered lipid digestion, absorption, and metabolism; influence on the gut microflora; and regulation of oxidative stress and inflammatory responses. The dairy food matrix, a term used to describe how the macronutrients and micronutrients and other bioactive components of dairy foods are differentially packaged and compartmentalized among fluid milk, cheese, and yogurt, may dictate how each affects cardiovascular risk. Current evidence indicates consideration of dairy foods as complex food matrices, rather than delivery systems for isolated nutrients, such as saturated fatty acids, is warranted.

**Statement of Significance**

This review, the first to describe the current potential mechanisms underlying beneficial effects of whole-milk dairy foods on risk markers for cardiometabolic health, challenges conventional thinking that whole-milk dairy foods, such as milk, cheese, and yogurt should be excluded from healthy dietary patterns.

## Introduction

Cardiovascular disease (CVD) is the leading cause of death globally [1]. Approximately 19 million deaths in 2020 were attributed to CVD, an increase of 18.7% from 2010 [1], with almost 80% occurring in low- and middle-income countries [2]. Lifestyle modification, including adherence to a healthy dietary pattern, is one strategy used to mitigate CVD. The Dietary Guidelines for Americans recommend consumption of low-fat and fat-free dairy foods, including milk, yogurt, and cheese, as part of overall healthy dietary patterns that help meet nutrient recommendations while keeping within recommended calorie and saturated fat limitations.

Cheese is the leading source of saturated fat in the American diet [3], and whereas it is recommended that Americans consume <10% of energy from saturated fat, data from NHANES 2015–2016 indicated 11.9% of calories in the American diet came from saturated fatty acids [4]. A body of observational and clinical evidence indicates, however, that whole-milk dairy food consumption is not associated with CVD, despite saturated fat content [5]. For the purposes of this article, whole-milk dairy foods were defined as those that were not low-fat (1% milk fat) or fat-free (skimmed, 0% milk fat), but included reduced-fat (2% milk fat) and whole (3.25% milk fat) milk, fermented milk, yogurt, and cheese—foods that are not recommended by the Dietary Guidelines for Americans because of their fat content, but contain bioactive fatty acids and have been demonstrated to have beneficial effects on cardiometabolic health. The aim of this review article is to describe the proposed mechanisms underlying inverse associations between whole-milk dairy food consumption and risk markers for cardiometabolic health.

## Methods

Articles referenced in this narrative review were obtained by searching PubMed and Google Scholar for relevant, peer-reviewed, full-text articles that were published in English since the year 2000. The search terms used included [milk OR cheese OR yogurt OR dairy OR whole milk dairy OR milk fat OR saturated fat OR milk fat globular membrane OR branched chain fatty acids OR odd chain fatty acids OR short-chain fatty acids OR milk polar lipids OR fermented dairy OR bioactive peptides OR dairy matrix] AND [cardio∗ OR coronary heart disease OR athero∗ OR blood pressure OR hypertension OR dyslipidemia OR inflam∗]. References from selected articles were scanned for relevance and inclusion.

### Milk fat is the most complex naturally occurring fat in a food

**FIGURE 1:**
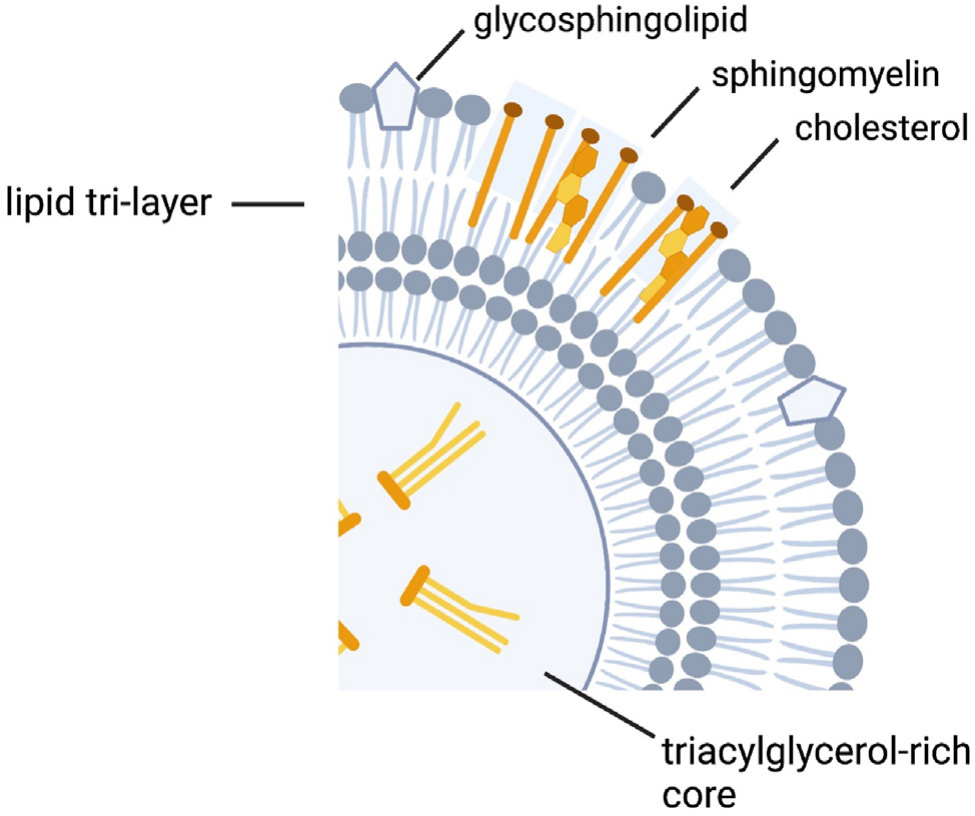
The milk fat globular membrane (MFGM) is unique in that it is a trilayer membrane that surrounds the lipids (triacylglycerols) of the milk fat globule core. The MFGM is composed of phospholipids and sphingolipids, referred to as milk polar lipids. Figure created with BioRender.com.

The lipids in bovine milk exist primarily as globules, known as milk fat globules [6]. Milk fat globules, comprised primarily of triglycerides [6], are coated with proteins and polar lipids that when secreted from the mammary gland are enveloped with the plasma membrane of the cell known as the milk fat globular membrane (MFGM; Figure 1) [6]. The MFGM is comprised of ∼70% proteins, many of which are enzymes, and 30% lipids, of which ∼25% are phospholipids, 3% are cerebrosides, and 2% are cholesterol [6].

Milk fat contains over 400 unique fatty acids, making it the most complex naturally occurring fat in a food [6]. In the United States, whole milk contains 3.25% milk fat [7], of which approximately two-thirds is saturated and one-third is unsaturated, including monounsaturated and polyunsaturated fatty acids [8]. Milk fat contains short-, medium-, and long-chain fatty acids that range in length from 4 to 24 carbons, with palmitic acid (C16:0) and stearic acid (C18:0) representing the majority of total saturated fatty acids (44.1% and 18.3%, respectively), and oleic acid (C18:1) predominating total unsaturated fatty acids (71%) [6]. Milk fat also contains unique fatty acids, such as branched-chain fatty acids (BCFAs) and the odd-chain fatty acids pentadecanoic acid (C15:0) and heptadecanoic acid (C17:0), saturated fatty acids that are produced by rumen bacteria [6].

### Potential mechanisms by which milk fat may beneficially affect cardiometabolic health

#### Milk polar lipids present in the MFGM may contribute to cardiometabolic health by mitigating plasma and hepatic hyperlipidemia

**FIGURE 2:**
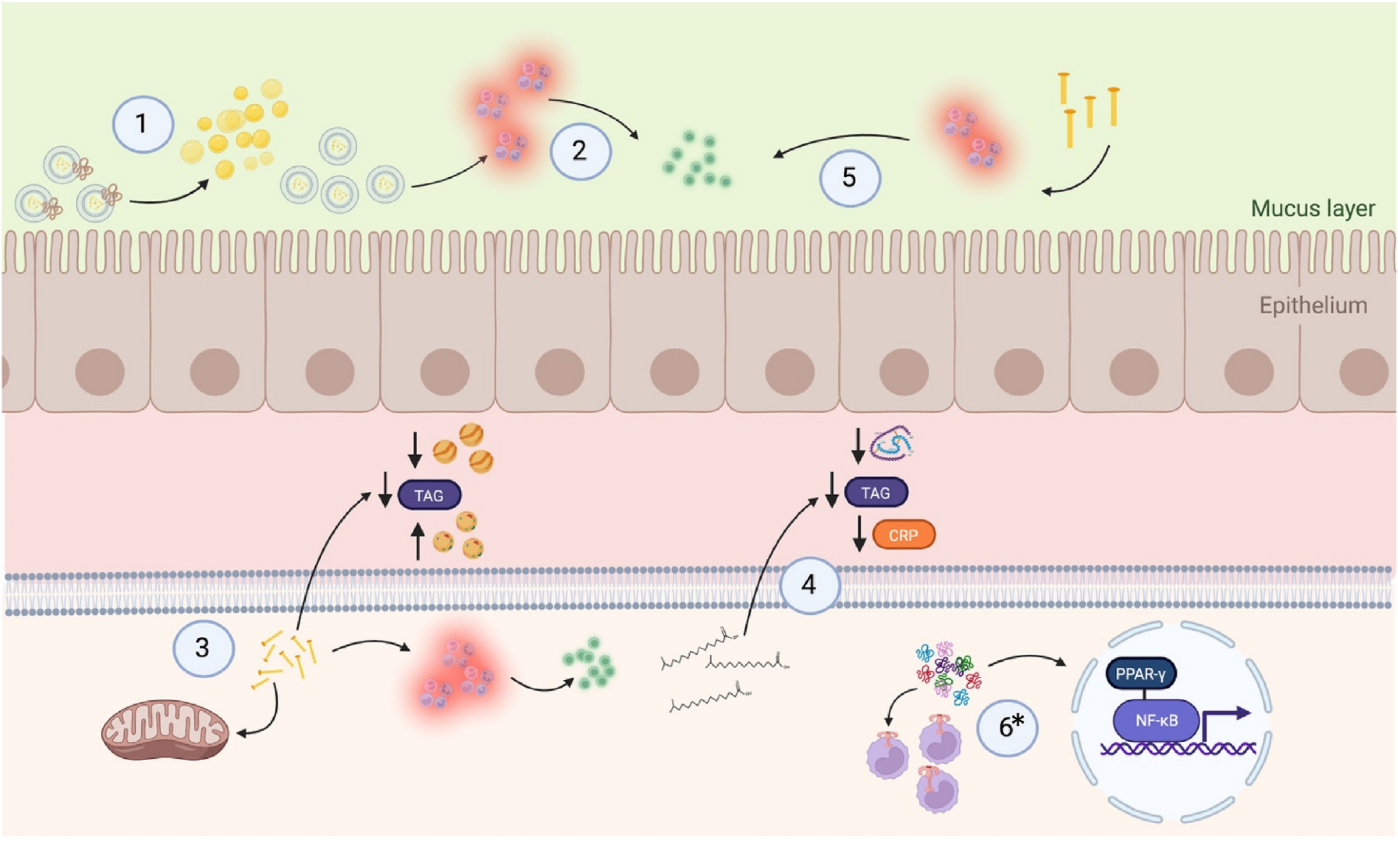
*1*) The milk polar lipids present in the milk fat globular membrane may improve cardiometabolic health by alleviating plasma and hepatic hyperlipidemia through reduced intestinal cholesterol absorption attributable to intraluminal emulsification [11] and the ability of milk sphingomyelin to bind pancreatic colipase, inhibiting the action of pancreatic lipase activity [12]; *2*) Milk polar lipids may beneficially affect cardiometabolic health by mitigating inflammatory responses in the gastrointestinal tract [11]; *3*) Results from observational studies in humans and preclinical trials in cell culture and animals indicate C15:0 and C17:0, odd-chain fatty acids present in milk fat, have beneficial effects on cardiometabolic health by improving dyslipidemia, stimulating mitochondrial repair, and reducing inflammation [13]; *4*) Total serum iso–branched-chain saturated fatty acids were inversely correlated with serum insulin, triglycerides, and C-reactive protein (CRP) concentration, indicating potential beneficial effects of iso–branched-chain saturated fatty acids on cardiometabolic health [14]; *5*) Preclinical research indicates short- and medium-chain saturated fatty acids may attenuate metabolic stress and inflammation via effects on the gut–brain axis [15]; *6*) A body of preclinical research demonstrated that milk-derived bioactive peptides may attenuate atherosclerotic plaque formation by inhibiting the NF-κB pathway in a peroxisome proliferator–activated receptor (PPAR) γ dependent manner, and by modifying the expression of C-C motif chemokine receptor 2 and toll-like receptor 4 receptors in monocytes [16]. Figure created with BioRender.com. ∗Mechanism applies to low-fat and fat-free dairy foods as well. NF-κB, nuclear transcription factor κB.

The MFGM is unique in that it is a trilayer membrane that surrounds the lipids of the milk fat globule core. The MFGM is composed of milk polar lipids (MPLs), phospholipids, and sphingolipids, which represent 2 of the most prominent components of the MFGM [9]. MPL account for ∼1% of milk fat with nearly equal thirds from sphingomyelins, phosphatidylcholines, and phosphatidylethanolamines [10]. Whereas the churning of milk fat disrupts the MFGM, resulting in the coalescence of free fatty acids that yield butter, the MFGM is present in most other whole-milk dairy foods and is particularly high in buttermilk [9]. Commercial processing of milk to manufacture low-fat and fat-free dairy foods results in the removal of the MFGM and significantly reduces total MPL by ≤40% [9]. Thus, whole-milk dairy foods, with exception to butter, are rich in MFGM and low-fat and fat-free dairy foods are not (Figure 2).

The MPL present in the MFGM may improve cardiometabolic health by alleviating plasma and hepatic hyperlipidemia through reduced intestinal cholesterol absorption attributable to intraluminal emulsification [17] and the ability of milk sphingomyelin to decrease the active concentration of cholesterol monomers in mixed micelles and bind pancreatic colipase, inhibiting the activity of pancreatic lipase [18]. Dietary polar lipids are natural emulsifiers in foods, and the chemical composition of MPL, which contains higher ratios of very-long-chain fatty acids (C22:0–C24:0) to palmitic acid (C16:0) and a varied distribution of sphingoid bases (d16:0–d19:0) in milk sphingomyelins, make them particularly effective at limiting the absorption of cholesterol [9].

Much prior understanding of the effects of MPL present in MFGM on cholesterol absorption came from mechanistic, preclinical data derived from rodent studies. Milk sphingomyelin was demonstrated effective at limiting intestinal cholesterol absorption in vitro and in vivo utilizing Caco-2 cell lines, male C57L/J mice [19], and male Sprague-Dawley rats [20], respectively. The investigation in male C57L/J mice identified milk sphingomyelin as having a high affinity for cholesterol as demonstrated by its ability to mitigate intestinal cholesterol absorption by reducing micellar cholesterol solubilization intraluminally, decreasing intestinal cholesterol uptake, and thereby decreasing monomeric cholesterol concentrations [19]. An investigation in male Sprague-Dawley rats indicated that the strong inhibitory effect of milk sphingomyelin on cholesterol absorption may have been attributable to the higher degree of saturated and longer chain lengths of fatty acyl groups, which may have slowed the decrease in the rate of luminal lipolysis, micellar solubilization, and uptake of micellar lipids into enterocytes compared with egg sphingomyelin [20]. It was further demonstrated in preclinical mouse models that MPL also attenuated hepatic cholesterol and triglyceride accumulation via attenuation of intestinal lipid absorption [9]. An investigation in female Swiss mice gavaged with polar lipids demonstrated that when compared with soy polar lipids, which do not contain sphingomyelins, MPL significantly increased plasma triglycerides and nonesterified fatty acids after 1 h, but significantly decreased these markers after 4 h [21]. An in vitro observation of lipid digestion showed that triglycerides were hydrolyzed significantly more from mice fed MPL compared with those fed soy polar lipids in a static human digestion model [21]. The researchers that conducted these studies suggested postprandial lipemia in the mice was modulated by MPL, likely through differences in chylomicron assembly and triglyceride hydrolysis [21]. The results from these studies in rodents helped inform our understanding of MPL but should not be extrapolated to humans since the amount of MPL fed to rodents was much higher than what would be obtained from consuming whole-milk dairy foods.

In humans, an MPL-enriched milk formulation containing 975 mg sphingolipids of which 700 mg was sphingomyelin (2–3 times more sphingolipids than normal dietary intake) did not significantly affect fasting plasma lipids or lipoproteins compared with a control after 4-wk daily ingestion by 33 male and 15 female adults that were free from disease [22]. These nonsignificant results were noteworthy, however, because the control group did experience nonsignificant trends showing increases in triglycerides, LDL and HDL cholesterol, and apolipoprotein B concentrations, indicating that MPL may have attenuated the hyperlipidemic response [22]. Similarly, in an 8-wk, single-blind, randomized controlled isocaloric parallel-design trial, 40 g of milk fat per day as butter oil significantly raised total cholesterol, LDL cholesterol, apolipoprotein B–to–apolipoprotein A-I ratio, and non-HDL cholesterol compared with an MFGM test diet in 57 randomly assigned males and females living with overweight [23]. The results of these studies indicated that in contrast to milk fat without MFGM, whole-milk dairy foods that contained MPL did not impair the lipoprotein profile in humans.

In 2 clinical investigations designed to evaluate whether MPL impacted human intestinal lipid absorption, metabolism, microbiota, and associated markers of cardiometabolic health, it was demonstrated that MPL improved markers of cardiometabolic health by interacting with the gut to reduce intestinal cholesterol absorption without disrupting the gut microbiota [17]. The dietary intervention trial was a double-blind randomized trial with a parallel group design in postmenopausal females who were living with overweight. In this study, after a 1-wk run-in period that included 100 g of control cheese per day, participants were assigned to a 4-wk intervention period that included cheese with either three or 5 g of MPL or the control cheese that was devoid of MPL [17]. The acute MPL consumption trial was also a double-blind randomized trial but with a crossover design in 58 ileostomy patients without obesity or dyslipidemia [17]. In this study, subjects participated in 3 distinct days of testing separated by a washout period in which they consumed a breakfast including 1 of the 3 test cheeses [17]. At the conclusion of 4 wk of the dietary intervention trial, MPL significantly reduced fasting and postprandial plasma cholesterol, total cholesterol–to–HDL cholesterol ratio, and apolipoprotein B–to–apolipoprotein A1 ratio [17]. Furthermore, consumption of MPL increased fecal coprostanol loss without disrupting the gut microbiota [17]. Additionally, the acute trial demonstrated that ingestion of MPL decreased cholesterol absorption and increased cholesterol-ileal efflux [17]. Mechanisms of action were attributable to MPL reducing intestine-derived chylomicrons, intestinal cholesterol absorption by coexcretion with the sphingomyelin fraction, and increased cholesterol to coprostanol conversion by the gut microbiota [17]. It is not yet understood, however, whether MPL directly impacted intestinal chylomicron secretion, hepatic clearance, and hypertriglyceridemia. Hence, why more research in this area is warranted.

Another double-blind randomized controlled 4-wk study assessed the impact of buttermilk consumption on plasma lipids and surrogate markers of cholesterol homeostasis in 15 males and 19 females with serum LDL cholesterol <5 mmol/L at screening; the consumption of 45 g/d of buttermilk for 4 wk resulted in significantly lower plasma total cholesterol, LDL cholesterol, and triacylglycerol compared with placebo [24]. Variations in β-sitosterol concentrations were significant predictors of LDL cholesterol response to buttermilk, indicating buttermilk consumption was associated with reduced cholesterol concentrations through inhibition of intestinal cholesterol absorption [24]. Furthermore, a double-blind randomized trial conducted in 62 and 57 males with overweight or obesity indicated that consumption of milk enriched with either 2 or 3 g daily of milk phospholipids, respectively, for 7 d resulted in increased activity of γ-glutamyl transferase, a marker of fatty liver disease, compared with milk enriched with either milk fat or soy phospholipids [25]. Collectively, these data support what has been demonstrated in rodent models; MPL may inhibit chylomicron assembly, thereby increasing chylomicron particle size and enhancing clearance [9,18,21]. The results of these studies indicated regular consumption of MPL may beneficially affect cardiometabolic risk.

The amount of MPL required to elicit effects in humans is not well understood, and considering MPL are detectable in low-fat dairy foods, albeit in much lower amounts than in whole-milk dairy foods, it is an area of future research that would be useful in determining if it could be detrimental to avoid all dairy fat in the diet. The aforementioned trials utilized 3–5 g/d of MPL; an amount that exceeds regularly reported consumption from commonly consumed dairy foods and yielded no detrimental effects on participants. Rather, neutral effects, meaning ones that did not impair cholesterol homeostasis, or beneficial effects, meaning improvements were reported in humans that consumed whole-milk dairy foods supplemented with MPL [17,[22], [23], [24]]. Current food-based recommendations call for 3 servings of low-fat and fat-free dairy foods daily. Results from these studies, however, indicate consumption of 2–3 servings of whole-milk dairy foods daily would not be detrimental to human health, and in some cases, may attenuate hyperlipidemia when compared with those who consume only low-fat and fat-free varieties of dairy foods. In fact, in a clinical trial conducted in 72 males and females, aged 18–75 y, living with metabolic syndrome, the consumption of 3.3 servings of whole milk, yogurt, and cheese for 12 wk had no effect on fasting serum total, LDL, and HDL cholesterol; triacylglycerides (TAG); or free fatty acids compared with participants who consumed diets low in dairy foods or rich in low-fat and fat-free dairy foods [26].

#### MPLs present in the MFGM may contribute to cardiometabolic health by reducing inflammatory responses to diet

It has been recognized that low-grade chronic inflammation of both innate and adaptive responses is involved in the development and progression of atherosclerosis, and remains a primary risk factor for CVD in high-risk patients even after desirable cholesterol levels are achieved [27]. Furthermore, it has been demonstrated that Western-style diets increase inflammatory responses and risk for chronic disease [28]. The observed effects of Western-style diets on inflammation and chronic disease risk are in part attributable to increased gut barrier permeability and LPSs in circulation that result from dysbiosis in the gastrointestinal tract [28,29]. When compared with egg sphingomyelin, milk sphingomyelin derived from the MFGM significantly reduced serum LPS levels in C57BL/6J mice fed a high-fat diet for 4 wk [30]. In another study in which C57BL/6J mice were fed a high-fat diet for 10 wk, both egg and milk sphingomyelin reduced inflammation and markers of macrophage infiltration in adipose tissue [31]. More recently, it was demonstrated in a neonatal rat model that MPL derived from buttermilk significantly downregulated the expression of proinflammatory cytokines IL-6, IL-8, and TNF-α, and upregulated the anti-inflammatory cytokine IL-10 [32]. MPL also significantly reduced activation of the LPS-induced toll-like receptor 4 (TLR-4)/nuclear transcription factor κB (NF-κB) signaling pathway in this model [32]. Results from these preclinical investigations indicated that MPL fed in high doses above what would be obtained from whole-milk dairy foods in the diet beneficially affected cardiometabolic health by mitigating inflammatory responses in the gastrointestinal tract.

A preclinical investigation in male LDL receptor knock-out mice fed high-fat, high-cholesterol diets for 6 wk demonstrated that the addition of milk sphingomyelin to the diet resulted in not only significantly lower VLDL and LDL cholesterol and reduced inflammatory markers in serum, liver, adipose, and aorta, but also in significantly less atherosclerotic lesion development in both the thoracic aorta and the aortic root [33]. Furthermore, mice fed MPL had improved microbiota composition with greater *Bacteroidetes*, *Actinobacteria*, and *Bifidobacterium,* and lower *Firmicutes* in cecal feces [33]. Doses in these investigations were higher than what would be obtained from diet, which is why it is important to further test these mechanisms in human subjects.

Sphingolipids are a class of bioactive polar lipids that have been demonstrated to contribute to cardiometabolic health and homeostasis via inflammatory signaling pathways [34]. Dysregulated sphingomyelin metabolism, detectable by increased circulating ceramide species, such as C24:1 ceramide, has been correlated with poor cardiometabolic health [34]. These polar lipids are detectable in MPL, but the consumption of MPL has not been implicated in inflammatory responses. In a 4-wk randomized controlled trial in 58 postmenopausal females, daily consumption of cream cheese enriched with ≤5 g of MPL resulted in decreased atherogenic sphingomyelin and ceramide species, C24:1 ceramide, C16:1 sphingomyelin, and C18:1 sphingomyelin, respectively, in plasma and chylomicrons, indicating improved cardiometabolic health [35]. These reductions were observed in ileal efflux and feces, and were correlated with reductions in total cholesterol, LDL cholesterol, and apolipoprotein B levels in subjects, indicating an effect of MPL on gut metabolism associated with cardiometabolic health [35].

In a randomized, double-blinded, crossover trial, 36 males and females with overweight and obesity consumed either a high-fat diet enriched with palm oil, which has been associated with postprandial inflammation, or a high-fat diet enriched with both palm oil and dairy MFGM; the meal containing MFGM resulted in significantly lower total cholesterol, LDL cholesterol, and soluble intracellular adhesion molecule, a marker of the inflammatory response [36]. The MFGM-enriched high-fat diet also increased IL-10, an anti-inflammatory cytokine [36]. The results of this study indicated an anti-inflammatory effect from MFGM. Whereas preclinical studies in a neonatal rat model indicated MPL significantly decreased the activation of TLR-4/NF-κB signaling and thereby had anti-inflammatory effects [32], the results of this study could not be attributed specifically to MPL since the MFGM was intact.

#### Odd-chain saturated fatty acids derived from milk fat may attenuate dyslipidemia and inflammation associated with Western diets by regulating metabolism and repairing mitochondrial function

In humans, even chain fatty acids with carbon chain lengths between 2 and 26 carbons represent >99% of total fatty acid concentration in plasma, with only 4 significantly measurable odd-chain fatty acids including C15:0, C17:0, heptadecenoic acid (C17:1), and tricosanoic acid (C23:0) [37]. Both C15:0 and C17:0 are present in milk fat due to rumen microbial fermentation and microbial de novo lipogenesis and can be used as biomarkers of milk fat consumption [37]. C15:0 and C17:0 present in United States retail milk at levels of 0.89 ± 0.004 and 0.52 ± 0.002 g/100 g of fatty acids, respectively [38] can be used as biomarkers of milk fat consumption [37] and to help distinguish low-fat and fat-free dairy consumers from whole-milk dairy consumers [39].

To understand the potential role of C15:0 in cardiometabolic pathways, researchers conducted a series of preclinical in vitro and in vivo studies across a variety of human cell lines and rodents, respectively. In cell culture, C15:0 was demonstrated to be a dual, partial peroxisome proliferator–activated receptor (PPAR) α/δ agonist that could repair mitochondrial function [40]. Furthermore, C15:0 induced anti-inflammatory and antifibrotic pathways by reducing monocyte chemoattractant protein 1 (MCP-1) and secreted immunoglobulin G as well as Collagen I and plasminogen activator inhibitor 1, respectively [40]. In Sprague-Dawley rats, oral doses of C15:0 induced higher plasma levels of C15:0 and C17:0, indicating de novo elongation of C15:0–C17:0 within 8 h of ingestion [40]. When C15:0 was fed in low doses of 5 mg/kg of body weight to C57BL/6J mice with obesity fed a high-fat diet for 90 days, circulating levels of proinflammatory chemokine, MCP-1, and IL-6 were all reduced compared with controls [40]. In New Zealand white rabbits fed a high-fat, high-cholesterol diet for a 2 wk run-in period, high daily supplementation of 35 mg/kg body weight of C15:0 for 11 wk resulted in lower cholesterol, triglycerides, globulins, and platelets, and exhibited improved liver function and did not progress in liver disease compared with nonsupplemented diseased controls [40].

A nested case-control design within the large prospective European Investigation into Cancer–Norfolk trial examined the relationship between plasma phospholipid fatty acid concentration and incident coronary heart disease (CHD) in 2,424 males and females with incident CHD compared with 4,930 controls, and found that compared with bottom quartiles, C15:0 and C17:0 concentrations were significantly inversely associated with CHD, reducing odds for the disease by 27% at 13 y of follow-up [41]. In a cross-sectional analysis of the European Investigation into Cancer–Potsdam study, the association between C15:0 and C17:0 and biomarkers of dyslipidemia were investigated in 1,759 males and females with varying degrees of dyslipidemia, overweight or obesity, and the prevalence of CVD, diabetes, and cancer [42]. In females, C15:0 in erythrocyte membranes was positively associated with plasma concentrations of HDL cholesterol, and C17:0 was inversely associated with triglycerides, indicating a beneficial association between C15:0 and C17:0 and biomarkers of dyslipidemia [42]. Conversely, in males, C17:0 in erythrocyte membranes was inversely associated with plasma HDL cholesterol [42], indicating potential differences in effects based on sex. In a cross-sectional study among 482 Japanese male and female adults free from disease, serum fatty acid levels of C15:0 and C17:0 were inversely associated with plasminogen activator inhibitor 1 [43]. The results of these studies indicated a potential role for dairy-derived odd-chain fatty acids, C15:0 and C17:0, in promoting cardiometabolic health perhaps by attenuating dyslipidemia, stimulating mitochondrial repair, and reducing inflammation. Currently, based on the evidence, some researchers are suggesting that C15:0 may in fact be an essential fatty acid [40]. C15:0 and C17:0 have also been proposed as biomarkers of fish consumption [44], and the possibility for misclassification cannot be ignored when utilizing biomarkers as indicators for food consumption.

#### BCFAs derived from milk fat may attenuate dyslipidemia and inflammation

Milk fat is a dietary source of BCFA, a class of fatty acids primarily derived from rumen bacteria with one or more methyl branches along the carbon chain [45]. Whereas endogenous synthesis pathways have been identified, consumption of dietary BCFA from dairy foods and ruminant meat is believed to be the primary source of BCFA in humans [45].

The effects of BCFA on inflammatory pathways have been investigated in human cell lines. In Caco-2 human epithelial cells treated with BCFA and activated with LPS, both anteiso- and iso-BCFA reduced IL-8 and expressed less TLR-4 compared with controls, indicating a reduction of proinflammatory markers [46]. A follow-up study utilizing vernix-monoacylglycerides enriched with 30% BCFA again demonstrated a reduction in IL-8 and TLR-4 as well as NF-κB [47]. These studies indicated BCFA reduced LPS-induced production of NF-κB and TLR-4 at the transcription level. These preclinical studies support the epidemiological observations that BCFA may beneficially affect health through anti-inflammatory pathways.

In a case-control analysis of 23 adult female bariatric surgery patients, patients with obesity had lower serum iso-BCFA when compared with controls without obesity [48]. Furthermore, total serum iso-BCFA was inversely correlated with serum insulin, triglycerides, and C-reactive protein (CRP) concentration, indicating potential beneficial effects of iso-BCFA on cardiometabolic health [48]. More research specific to whole-milk dairy food sources of BCFA is warranted.

#### Short- and medium-chain fatty acids derived from milk fat may attenuate metabolic stress and inflammation

Short- and medium-chain saturated fatty acids comprise ∼5% and 8% of total fatty acids in cow’s milk, respectively [49]. Butyric acid (C4:0) has been identified as a short-chain fatty acid that may modulate immunity. Preclinical research on butyric acid in rodents indicated a role in appetite reduction and fat oxidation via effects on the gut–brain axis [50]; triglyceride hydrolysis, free fatty acid oxidation, and attenuated inflammation in adipose tissue through effects on specific G-protein coupled receptors and gut microbiota composition [51]; and slowed the progression of atherosclerosis through actions related to the reduction of proinflammatory cytokines and lower activation of the NFκB pathway [52]. Short-chain fatty acids were also determined to be effective modulators of PPARγ in a preclinical study in mice in which it was demonstrated that short-chain fatty acids decreased PPARγ expression and activity that led to the increased expression of mitochondrial uncoupling protein 2 and stimulation of oxidative metabolism in liver and adipose tissue [53].

In a human clinical trial in which 4 g of sodium butyrate was orally administered daily in 9 men living without disease and 10 men living with obesity and metabolic syndrome, and peripheral blood mononuclear cells were isolated before and after supplementation for use in experiments on immune function, butyrate was demonstrated to beneficially affect trained immunity in the monocytes of men with obesity with metabolic syndrome [54]. The oral dose of 4 g utilized in the study was far higher than that which would be provided by 3 servings of whole-milk dairy foods per day but lends insight into the potential mechanisms by which butyrate may contribute to beneficial effects on metabolism that are observed with the consumption of whole-milk dairy foods.

Medium-chain fatty acids (C8:0–C12:0) have also been implicated in the attenuation of inflammation and beneficial effects on energy homeostasis and metabolic health [49]. Whole-milk dairy foods contribute small amounts of short- and medium-chain fatty acids to the diet, and effects from natural consumption should not be confused with those from pharmacological doses [55], but short- and medium-chain fatty acids may contribute, in part, to beneficial effects on metabolism observed from consumption of whole-milk dairy foods [49].

#### The dairy food matrix may play a role in the effects on cardiometabolic health by influencing fat absorption and metabolism

Over the last century, dietary guidance has focused on food as a delivery system for nutrients that could mitigate deficiencies. For example, recommending dairy consumption meant providing calcium and vitamin D in the diet to help prevent the occurrence of nutritional rickets [56]. In the last half-century, however, dietary guidance in higher-income countries has evolved to address the twin epidemics of overweight and undernourishment that simultaneously coexist [57]. Now, dairy foods can be seen as a delivery system not only for calcium and vitamin D but also for fat, particularly saturated fat. Thus, current recommendations in the United States are to consume low-fat and fat-free dairy foods to meet nutrient goals while staying within calorie and saturated fat recommendations, which are to limit saturated fat to <10% of calories and replace with unsaturated fatty acids in the diet [58].

In a food-level modeling study of dietary nutrient density and diet quality that utilized data from 15,260 males and females aged 4 y and older who completed a 24-h dietary recall from the 2013–2016 NHANES, researchers assessed the implications of nutrient-for-nutrient swaps on dietary nutrient density and healthy diet scores after the removal of food sources of nutrients from the diet of Americans [59]. The health status of the population was not reported. Researchers found that removing 5% of dietary energy from dairy fat led to lower amounts of multiple micronutrients in the diet such as calcium, vitamin D, vitamin A, riboflavin, niacin, and vitamin B12 resulting in a lower nutrient-rich food score [59]. The study demonstrated that swapping dairy fat for foods containing nondairy monounsaturated and polyunsaturated fatty acids did alter fatty acid ratios in the diet, but not without significantly lowering key micronutrients in the diet [59]. These studies demonstrated the complexity of diets and the pitfalls of focusing on single nutrients. Simplifying the substitution of a single nutrient such as saturated fat without considering the food source of that substitution may have consequences on health.

Advances in nutrition science over the last quarter of a century indicate foods are complex matrices of macronutrients and micronutrients and other bioactive components that are differentially packaged and compartmentalized, which, in turn, affects how foods influence metabolism and disease [60,61]. Current evidence indicates dairy foods such as milk, cheese, and yogurt have differential effects on human health, and that associations between whole-milk dairy foods and cardiometabolic risk cannot be predicted by their total and saturated fat contents, which may in part be attributable to the differences in their complex food matrices.

The aforementioned mechanisms related to whole-milk dairy food consumption and effects on markers of cardiometabolic health discussed in this review were specific to dairy-derived fatty acids and milk fat. It cannot be ignored, however, that low-fat and fat-free dairy foods have also been associated with cardiometabolic health, which must be attributable to other biological mechanisms, perhaps related to the dairy food matrix. For example, studies in animals and humans indicate the dairy matrix may influence fat absorption and metabolism. In a parallel-designed, randomized, controlled study of 36 crossbred growing sows, diets containing regular-fat cheese raised total and HDL cholesterol compared with diets containing butter after 2 wk of feeding, an effect not observed in sows fed reduced-fat cheese [11]. Whereas both regular and reduced-fat cheese led to higher fecal fat excretion compared with butter, only regular-fat cheese resulted in fecal fat excretion that was correlated with lower *Firmicutes-*to-*Bacteroidetes* ratio in the microbiome, an indicator of a beneficial effect from cheese [11]. The differential effects of butter, regular-fat and reduced-fat cheese observed in this study indicated an effect of the dairy matrix on metabolic health in growing pigs, with the greatest benefit observed in the full-fat cheese group. In a randomized controlled parallel-design trial conducted in 139 male and female human subjects with 2 or more risk factors for metabolic syndrome, however, there were no differences detected between regular-fat cheese, reduced-fat cheese, or control diet on LDL cholesterol, triglycerides, or waist circumference after 12 wk of study [12]. Whereas the results of this study did not yield significant effects, it also did not show a detriment to consuming regular-fat cheese or a benefit to consuming reduced-fat cheese.

Two other clinical trials in humans, however, did establish an association between increased fecal fat excretion and attenuated blood lipid response after the consumption of dairy foods that were rich in calcium [62]. In a randomized crossover study designed to test the influence of calcium on the effects of high-fat diets on blood lipids in 15 male subjects free from disease, it was demonstrated that high- compared with low-calcium diets resulted in a decrease in total cholesterol–to–HDL cholesterol ratio and an increase in HDL cholesterol–to–LDL cholesterol ratio [63]. In both high-calcium and high-fat diets, fecal fat excretion was increased [63]. The results of this study indicated that calcium in dairy attenuated the rise in total and LDL cholesterol induced by regular-fat cheese, without reducing HDL cholesterol. After this investigation, another randomized, crossover study investigated whether milk- and cheese-based diets with similar calcium contents differentially affected saturated fatty acid–induced increases in blood lipids in 15 healthy males [64]. After 2 wk of consumption, a diet that included cheese and reduced-fat milk and was adequate in calcium with 1700 mg/d, increased total cholesterol and LDL cholesterol to a lesser extent than a nondairy diet with approximately just 500 mg of calcium per day [64]. Fecal fat excretion was also higher after consumption of the reduced-fat milk and cheese diets than the nondairy diet [64]. The results of this trial demonstrated that diets that contained reduced-fat milk (2% milk fat), regular-fat cheese, and adequate amounts of calcium increased fecal fat excretion and attenuated the effects of saturated fat on total and LDL cholesterol levels when compared with a nondairy diet low in calcium. These trials highlighted that the effects of foods on metabolism cannot be predicted based on single nutrients, but rather taken altogether for the complex matrices that they are.

The mechanism by which calcium-rich whole-milk dairy foods may attenuate the blood lipid response may also be attributable, in part, to the phosphorus contained in those foods [62]. It is known that dietary calcium and phosphate precipitate in the small intestine and form insoluble amorphous calcium phosphate that can bind to luminal bile acids [65]. A placebo-controlled, double-blind crossover study in 31 males and females in their mid-20s, free from disease, that tested whether amorphous calcium phosphate bound to luminal bile acids affected cholesterol metabolism demonstrated that amorphous calcium phosphate lowered serum total cholesterol through an increased excretion of bile acids that resulted in increased de novo bile acid synthesis [65]. Taken together, the neutral effects of whole-milk dairy foods on plasma lipids associated with cardiometabolic disease may be attributable to matrix effects on lipid metabolism, the microbiome, and fecal fat excretion.

#### The fermented dairy matrix may attenuate dyslipidemia and chronic inflammation through interactions with the gut microbiome

Studies in animals and humans indicate fermented dairy foods may beneficially affect blood lipid concentrations [66] and attenuate markers of chronic inflammation [62]. In a randomized, controlled, crossover design study conducted in 43 male and female adults, free from disease, aged 18–65 y, firm cheddar cheese, soft cream cheese, and butter had differential effects on plasma concentrations of triglycerides at 2 and 6 h after consumption, with cheddar cheese eliciting the lowest TAG response at 2 h, but the TAG response from cream cheese being most attenuated at 6 h, demonstrating the cheese matrix modulated the impact of dairy fat on postprandial lipemia in healthy adults [67]. The overall triglyceride response, however, did not differ among groups [67]. In a larger, parallel-design study of 164 male and female adults aged 50 y and older and living with overweight, the effect of the cheese matrix on blood lipids was tested over 6 wk by comparing cheddar cheese, reduced-fat cheddar cheese plus butter, butter alone, or a calcium caseinate powder plus calcium supplement as delivery systems for ∼40 g of dairy fat per day [68]. Results from the trial demonstrated total cholesterol and LDL cholesterol were lower when fat was contained within the cheese matrix, as delivered by the cheddar cheese, compared with butter [68]. Results from a randomized, controlled, crossover trial conducted in 18 healthy men demonstrated postprandial triglycerides and free fatty acids increased after consumption of a micellar casein isolate gel compared with cheddar or homogenized cheese indicating an influence from casein networks on lipid responses [69]. This aligned with an earlier in vitro investigation by the same lab group that demonstrated a significantly lower release of free fatty acids from cheese compared with micellar casein isolate products, indicating the cheese matrix resisted digestion resulting in large fat droplets that slowed the rate of lipid digestion [70].

A mechanism by which fermented dairy foods may beneficially affect blood lipid responses is by favoring the production of short-chain fatty acids by the gut microbiota [71]. In a crossover trial that utilized nuclear magnetic resonance–based metabolomics to investigate the association between cheese or milk consumption and blood cholesterol levels in 15 male young adults free from disease, cheese consumption increased fecal butyrate, propionate, and malonate and decreased fecal acetate and glycerol concentrations compared with control [71]. Furthermore, a significant inverse correlation between fecal short-chain fatty acids and LDL cholesterol concentrations indicated a link between fermented dairy consumption, the gut microbiome, and cholesterol metabolism [62,71].

Gut dysbiosis is associated with increased bacterial LPS concentration, which promotes inflammation and cardiometabolic disease [72]. The links between fermented dairy foods, the gut microbiota, and cardiometabolic risk may also be attributable to a reduction in LPS-induced inflammation by improving gut barrier permeability, a mechanism observed utilizing probiotic strains in murine models [73], but yet to be confirmed in humans [74]. An aforementioned preclinical investigation in pigs demonstrated that low- compared with regular-fat fermented dairy foods had differential effects on the gut microbiota with regular-fat cheese leading to a lower *Firmicutes*-to-*Bacteroidetes* ratio, indicative of beneficial effects of cheese on the microbiome [11]. Lactate produced from lactic acid-producing bacteria during fermentation may reduce the production of reactive oxygen species in enterocytes by mitigating proinflammatory cytokine production [75]. Another preclinical study conducted in 8-wk old LDL receptor/apolipoprotein B100 knock-out mice demonstrated that both fermented milk protein and fermented yogurt protein upregulated the *Streptococcus* genus, and decreased circulating adhesion molecules as measured by vascular cellular adhesion molecule 1 and intracellular adhesion molecule 1 compared with nondairy protein and milk protein controls after 12 wk of feeding [76]. Furthermore, yogurt protein increased the expression of genes involved in intestinal immunity and integrity compared with nondairy protein, and fermented milk protein attenuated hepatic inflammation as measured by MCP-1, IL1-β, and interferon γ compared with nondairy protein and milk protein controls [76]. The results of this study indicated an effect of fermentation on the ability of dairy protein products to mitigate markers of inflammation possibly through modulating the gut microbiota in diet-induced mice with obesity [76].

In an investigation of the relationship between fermented dairy food consumption and the intestinal microbiota and inflammatory response among 130 male and female middle-aged adults that were free from disease, unsweetened yogurt consumers showed increased fecal levels of *Akkermansia*, and sweetened yogurt consumers showed decreased levels of *Bacteroides* compared with yogurt nonconsumers [77], indicating beneficial effects of yogurt consumption on the gut microbiome. Yogurt consumption was also associated with lower circulating CRP, a marker of chronic inflammation [77]. A randomized, controlled, crossover study conducted in 47 male and female adults aged 18–70 y who consumed high-fat meals composed of butter, cheese, whipped cream, or sour cream demonstrated that the consumption of fermented dairy foods, particularly cheese, induced a less inflammatory postprandial response, as measured by peripheral blood mononuclear cell expression of inflammation-related genes [78]. Furthermore, the consumption of cheese increased plasma amino acid concentrations that were correlated with anti-inflammatory gene expression [78].

#### The fermented dairy matrix may promote antihypertensive effects through angiotensin-converting enzyme-inhibitory activity in both fat-containing and fat-free dairy foods

Fermentation of milk increases the production of bioactive peptides from milk proteins that are normally released during digestion [72]. Bioactive tripeptides valine-proline-proline and isoleucine-proline-proline from fermented dairy foods may inhibit angiotensin-converting enzyme (ACE) activity leading to a reduction in blood pressure [72,79]. In a randomized, parallel-design study of 94 hypertensive male and female adults in their mid-fifties, a fermented milk containing 7.5 mg/100 g of isoleucine-proline-proline tripeptide and 10 mg/100 g of valine-proline-proline tripeptide resulted in a significant reduction in systolic and diastolic blood pressure after 4 wk compared with control [80]. Whereas the absorption of bioactive peptides has been recorded in rats [81], more research is needed to elucidate the exact mechanisms by which bioactive peptides may inhibit ACE activity in humans.

Results from clinical trials over the years are varied based on the fermented milk product and the strain used in fermentation. Whereas a Cochrane review of 15 randomized controlled trials concluded that fermented milk lowered systolic blood pressure, but not diastolic blood pressure [82], another meta-analysis of 13 trials concluded that fermented milk lowered both systolic and diastolic blood pressure compared with controls, and with a greater effect in hypertensive subjects [13]. Taken together, data indicate fermented milk has the potential to offer antihypertensive effects pending its formulation. Whereas bioactive peptides can be contained in low-fat and fat-free dairy foods, Americans consume the majority of fermented dairy as whole-milk cheese [83].

#### The cheese matrix may help improve microvascular function through increased production of bioactive peptides

A body of preclinical research demonstrated that milk-derived bioactive peptides may attenuate atherosclerotic plaque formation by inhibiting the NF-κB pathway in a PPARγ-dependent manner, and by modifying the expression of C-C motif chemokine receptor 2 and TLR-4 receptors in monocytes [14]. Whereas these mechanisms have been demonstrated in vitro and in experimental rodent studies, randomized clinical trials are necessary to elucidate the effects of bioactive peptides derived from the consumption of whole-milk dairy foods.

Bioactive peptides have also been demonstrated to protect the microvasculature with their antioxidant properties [84]. Whereas dietary sodium is associated with oxidative stress and impaired microvascular function, cheese may have beneficial effects on the microvasculature, despite its sodium content [84,85].

In a randomized, controlled trial conducted in 14 healthy males and females, aged 61 ± 2 y, given 85 g of either dairy cheese, soy cheese, or pretzels containing 560 mg of sodium; 170 g of dairy cheese; or 130 g of pretzels containing 1,120 mg of sodium, nitric oxide-dependent vasodilation was greater after consumption of dairy cheese when compared with soy cheese or pretzels [84]. Infusion with ascorbate, a potent antioxidant, promoted nitric oxide-dependent vasodilation after soy cheese or pretzel consumption, but not dairy cheese, indicating that dairy cheese consumption mitigated sodium-induced microvascular dysfunction through antioxidation [84]. In a randomized, controlled, crossover intervention conducted over 8 d in 11 adults aged 55–75 y and free from disease, the incorporation of cheese preserved endothelium-dependent dilation by decreasing the concentration of superoxide radicals compared with high-sodium diets without cheese [85].

## Discussion

Evidence from a body of literature published over the last 2 decades indicates that total self-reported dairy consumption does not increase the risk for CVD (Table 1). A limitation to these findings is that these data are taken from dietary questionnaires, which are subject to random and systematic measurement errors including, but not limited to recall bias, underreporting, and the misclassification of individuals and foods. This is important to keep in context when summarizing results from observational studies.

**TABLE 1:** Meta-analyses of prospective cohort studies that investigated the relationship between dairy and cardiovascular diseases

Reference	Study design and objective	Population	Results	Risk
Jakobsen et al. [86] 2021	SR and dose-response MA of PCS to summarize findings on the associations between total and subgroup dairy product intake and risk of major atherosclerotic CVD	General adult population	Total dairy, total high-fat dairy and total low-fat dairy were not associated with risk of CHD or ischemic stroke. Total milk and low-fat milk were not associated with risk of CHD. High-fat milk was positively associated with CHD, RR: 1.08 (95% CI: 1.00, 1.16); *I*^2^ = 0%; *P*_*het*_ = 0.94/200 g higher intake per day. Total milk was inversely associated with ischemic stroke, RR: 0.88 (95% CI: 0.79, 0.98); *I*^2^ = 0%; *P*_*het*_ = 0.52. Yogurt was not associated with risk of CHD or ischemic stroke. Cheese was inversely associated with CHD, RR: 0.96 (95% CI: 0.93, 0.98); *I*^2^ = 3%; *P*_*het*_ = 0.41/20 g higher intake per day. Cheese was not associated with risk ischemic stroke	
Chen et al. [87] 2021	SR and MA of PCS to synthesize evidence on the associations between dairy consumption and risk of HTN, CHD, and stroke	General adult population	Total dairy associated with lower risk of HTN, RR: 0.91 (CI: 0.86, 0.95), *I*^2^ = 73.5%, CHD, RR: 0.96 (CI: 0.92, 1.00), *I*^2^ = 46.6%, and stroke, RR: 0.90 (CI: 0.85, 0.96), *I*^2^ = 60.8%	
Zhang et al. [88] 2020	MA of cohort studies to determine the association between fermented dairy food intake and risk of CVD	General adult population	Fermented dairy was inversely associated with odds of CVD, OR = 0.83 (CI: 0.76, 0.91). Cheese, OR = 0.87 (CI: 0.80, 0.94). Yogurt, OR = 0.78 (CI: 0.67, 0.89)	
Bechthold et al. [89] 2019	SR and MA of PCS to synthesize knowledge about the relationship between major food groups and risk of CHD, stroke, and HF	Adult population free from chronic disease	No associations were detected for total dairy and risk of CHD, stroke, or HF. Additional 200 g of dairy per day were positively associated with risk of HF, RR: 1.08 (95% CI: 1.01, 1.15). Increasing dairy up to ∼500 g/d was associated with ∼5% decreased risk of stroke	
Mishali et al. [90] 2019	SR and MA of PCS to examine whether dairy consumption has different effects on T2D and CVD in men and women	General adult population	Total dairy associated with reduced risk for T2D, RR: 0.868 (CI: 0.82, 0.92), *P* < 0.001, and CVD, RR: 0.837 (CI: 0.75, 0.93), *P* < 0.001, in women, not men	
Guo et al. [91] 2017	Dose-response MA of PCS to summarize findings on the associations between total and subgroup dairy product intake and risk of all-cause mortality, CHD and CVD	General adult population	No associations were detected for total dairy, total high-fat dairy, total low-fat dairy, fermented dairy, cheese, and yogurt and risk for all-cause mortality, CHD or CVD	
Chen et al. [92] 2017	MA of PCS to evaluate the risks of total CVD, CHD, and stroke associated with cheese	General adult population	Cheese consumption was associated with reduced risk of CVD, RR: 0.90 (CI: 0.82, 0.99); CHD. RR: 0.86 (CI: 0.77, 0.96); and stroke, RR: 0.90 (CI: 0.84, 0.97)	
De Goede et al. [93] 2016	SR and dose-response MA of PCS to determine relationship between dairy and risk of stroke	General adult population	Lower risk of stroke was associated with 200 g/d increments of milk, RR: 0.93 (CI: 0.88, 0.98), *P* = 0.004, *I*^2^ = 86% and 40 g/d of cheese, RR: 0.97 (CI: 0.94, 1.01)	
Alexander et al. [94] 2016	SR and dose-response MA of PCS to determine the association between dairy consumption and risk of CVD	General adult population	Total dairy was associated with reduced risk of stroke RR: 0.91 (CI: 0.83, 0.99). Cheese was associated with reduced risk of CHD, RR: 0.82 (CI: 0.72, 0.93) and stroke, RR: 0.87 (CI: 0.77, 0.99)	

Abbreviations: CHD, coronary heart disease; CI, confidence interval; CVD, cardiovascular disease; HF, heart failure; HTN, hypertension; MA, meta-analysis; OR, odds ratio; PCS, prospective, cohort study; RR, relative risk; SR, systematic review; T2D, type 2 diabetes.

There is substantial clinical evidence to support the observations that whole-milk dairy consumption does not increase the risk for CVD, despite its fat content. The Dietary Approaches to Stop Hypertension (DASH) diet, is a clinically tested dietary pattern demonstrated to lower blood pressure [95]. The standard DASH dietary pattern is a reduced-fat plan containing, daily, 8–10 servings of fruits and vegetables, 2–3 servings of low-fat and fat-free dairy, whole grains, poultry, fish, and nuts [95]. One randomized controlled trial examined the health effects of including whole-milk dairy foods in a modified DASH diet, which increased saturated fat from 8% to 14% of calories and total fat from 27% to 40% of calories, and reduced carbohydrates from 55% to 43% of calories when compared with the standard DASH diet among free-living middle-aged adults free from disease. Compared with the standard DASH diet, the modified DASH diet lowered blood pressure to the same degree, significantly reduced blood levels of triglycerides, and did not increase total cholesterol or LDL cholesterol, or decrease HDL cholesterol [96], which demonstrated that whole-milk dairy foods improved standard biomarkers related to CVD risk when incorporated into a healthy dietary pattern that was calorie-balanced. Body weight remained stable during the intervention, with no differences between diets. Of note, the displacement of carbohydrates from the diet was of importance since it was previously established through a well-controlled randomized controlled trial that dietary and plasma saturated fat were not related and that increasing dietary carbohydrate across a range of intakes stimulated increased plasma palmitoleic acid, a biomarker associated with adverse cardiometabolic outcomes [15]. These results demonstrated the necessity of food-related research to consider the total dietary pattern and perhaps shift focus away from dietary fat and consider the overconsumption of carbohydrates when fat is removed from the diet.

In another randomized controlled trial in 111 subjects designed to test the effects of dairy consumption and its fat content on glycemic control and risk factors for CVD in middle-aged male and female patients living with type 2 diabetes, participants were assigned to either a control group, instructed not to change any aspect of their diet, including dairy consumption; a low-fat dairy group, instructed to consume 3 or more servings of low-fat dairy foods consisting of <2% milk fat daily; or a high-fat dairy group, instructed to consume 3 or more servings of whole-milk dairy foods with 2% milk fat or higher, daily for 24 wk [97]. After 24 wk of study, there were no statistically significant differences in glycated hemoglobin (HbA1c) or body weight, and there were no significant changes from baseline in HbA1c, body weight, BMI, waist circumference, or waist-to-hip ratio among the 3 groups [97]. HbA1c is not only included in diagnostic criteria for type 2 diabetes, but it serves as an effective retrospective marker of sustained blood glucose control, since it is determined by the 90-d lifespan of the erythrocyte. Furthermore, there were no significant differences in mean changes in CRP, fasting glucose, HOMA-IR, or blood pressure between the 3 groups [97]. Whereas based on findings from observational studies, researchers had hypothesized increasing dairy consumption would result in beneficial decreases in markers of cardiometabolic health, in fact, no changes were detected [97]. Despite the increase in total energy intake from total and saturated fat in the high-fat dairy group compared with the other groups, there were no significant differences in mean changes in LDL or HDL cholesterol, triglycerides, or VLDL cholesterol from baseline to 24 wk among the 3 groups [97]. This trial demonstrated there were no differences in body composition, lipid parameters, or blood pressure in subjects consuming whole-milk dairy foods when compared with low-fat dairy foods after 6 mo of study [97].

When compared with no dairy consumption, consuming whole-milk dairy foods for 4 wk did not significantly affect blood pressure or vascular function among 60 adults in a crossover intervention conducted in males and females with elevated blood pressure [16]. In this trial, 4 daily servings of whole milk or whole-milk dairy foods were added to the normal diet and compared with a diet devoid of dairy for 4 wk separated by a 2-wk washout period [16]. At the conclusion of the study, there were no significant differences detected in carotid-femoral pulse wave velocity, vascular distension measures measured by ultrasound, or brachial arterial flow-mediated dilation during the high dairy and no dairy interventions [16]. The participants maintained body mass throughout the study. The results of this study indicated that the addition of whole-milk dairy foods to the habitual diet had no effect on subclinical vascular function in adults with high blood pressure. Another randomized trial, conducted in 66 subjects—adult males and females with metabolic syndrome—who, after a 4-wk run-in period in which they limited their dairy consumption to 3 or less servings/wk of fat-free milk, were randomly assigned to either continue with that treatment of limited dairy consumption or switch to a diet containing 3.3 servings per day of either low-fat or whole milk, cheese, and yogurt for 12 wk showed there were no intervention effects on fasting serum total, LDL, and HDL cholesterol, triglycerides, or free fatty acids or diastolic or systolic blood pressure among participants [26]. The switch to a diet containing dairy foods resulted in increased waist circumference and body weight, which researchers attributed to increased caloric intake. This study demonstrated that a diet rich in whole-milk dairy foods had no effects on traditional risk factors for CVD in adults with metabolic syndrome. Moreover, a meta-analysis of 18 randomized controlled trials conducted around the world found that total consumption of milk, cheese, and yogurt had no significant unfavorable effects on lipids, including total, LDL or HDL cholesterol, or triglycerides among male and female adults [98]. Considered altogether, the results from a body of clinical research indicate that whole-milk dairy foods, despite their saturated fat content, may attenuate risk for CVD through multiple pathways that affect lipid and cholesterol metabolism, vascular endothelial function, and regulation of blood pressure.

Current dietary guidance encourages the consumption of low-fat and fat-free dairy foods, because of the saturated fats and calories contained in whole-milk dairy. Based on the available evidence, however, the proposed benefits of low-fat and fat-free dairy foods may actually be associated with the dairy matrix [99]. Americans currently fall short of recommended dairy consumption [58], and it is not known if closing the gap with whole-milk dairy foods would actually be detrimental to cardiometabolic health. In fact, current literature indicates that whole-milk dairy food consumption does not increase the risk for cardiometabolic disease [100], and could even help reduce the risk for several metabolic disorders.

## Conclusion

The dairy food matrix, how the macronutrients and micronutrients and other bioactive components of dairy foods are differentially compartmentalized among fluid milk, cheese, and yogurt, may dictate how each affects cardiovascular risk. Dairy fatty acids and polar lipids, within the MFGM, are part of the dairy matrix of whole-milk dairy foods. As such they may also contribute to cardioprotective benefits through various complex pathways involving lipid digestion, absorption, and metabolism; the gut microbiome; and oxidative stress and inflammatory regulation that may be influenced by processing. Therefore, reducing the health effects of dairy foods down to single nutrients is too simplistic a concept. For instance, current data indicate the MFGM present in fluid buttermilk may mitigate hyperlipidemia and inflammation. The fermented cheese matrix that includes calcium, protein, and bioactive milk fat has been demonstrated to beneficially affect blood lipids, attenuate markers of chronic inflammation, and increase the production of bioactive peptides. The fermented yogurt matrix may beneficially affect the gut microbiota and fermented milk may promote antihypertensive effects through ACE-inhibitory activity. These examples highlight the complexity of various whole-milk dairy foods and how they may differentially impact human health. Based on these findings, it is prudent to consider dairy as a complex food matrix, rather than a delivery system for isolated nutrients. Research to expand our knowledge of the effects of whole-milk dairy foods on human health, perhaps through nutritional genomics, is warranted at this time.

## Author contributions

The authors’ responsibilities were as follows – MT-G: designed the review; BHRB: conducted the review and wrote the manuscript; and both authors: read and approved the final manuscript.

## Conflict of interest

MT-G is Vice President of Nutrition Research for National Dairy Council. BHRB was compensated by National Dairy Council for her time preparing this review.

## Funding

This review was funded by National Dairy Council.
